# Influence of glycaemic management and BMI on cardiac autonomic markers in children with type 1 diabetes: a prospective cohort study

**DOI:** 10.1007/s00125-025-06592-3

**Published:** 2025-11-11

**Authors:** Ebba Bergdahl, Gun Forsander, Linda Milkovic, Frida Sundberg, Frida Dangardt

**Affiliations:** 1 Department of Molecular and Clinical Medicine, Institute of Medicine, Sahlgrenska Academy, University of Gothenburg, Gothenburg, Sweden; 2 Department of Paediatrics, Institute of Clinical Sciences, Sahlgrenska Academy, University of Gothenburg, Gothenburg, Sweden; 3 Department of Paediatric Clinical Physiology, Queen Silvia Children’s Hospital, Sahlgrenska University Hospital, Region Västra Götaland, Gothenburg, Sweden; 4 School of Medical Sciences, Örebro University, Örebro, Sweden; 5 Paediatric Heart Centre, Queen Silvia Children’s Hospital, Sahlgrenska University Hospital, Region Västra Götaland, Gothenburg, Sweden

**Keywords:** Autonomic dysfunction, Cardiac autonomic neuropathy, Children, Paediatrics, Type 1 diabetes

## Abstract

**Aims/hypothesis:**

Our aim was to examine the presence of subclinical cardiovascular autonomic neuropathy (CAN) in a cohort of children with well-regulated type 1 diabetes by measuring baroreceptor sensitivity (BRS), QT variability index (QTVI) and heart rate variability (HRV).

**Methods:**

Forty-five children (aged 6–15.99 years) with a type 1 diabetes duration of ≥5 years, and 37 healthy control children were included at baseline; and 28 and 18 children, respectively, were included at 2 year follow-up. Cardiac BRS, QTVI and HRV were measured and anthropometrical data and blood samples were collected from all study participants. Longitudinal HbA_1c_ values from 3 months after type 1 diabetes diagnosis and continuous glucose monitoring data from the children with type 1 diabetes were also collected.

**Results:**

Time in normoglycaemia (TING) increased significantly from 42% to 48% between baseline and 2 year follow-up (*p*=0.042). No difference in BRS, QTVI or HRV were found between the study groups at baseline or follow-up. Children with type 1 diabetes with a BMI *z* score ≥1 showed higher QTVI compared with either lean children with diabetes or healthy control children. QTVI correlated with type 1 diabetes duration, longitudinal HbA_1c_ AUC and cystatin C in children with type 1 diabetes at baseline, and with CV at follow-up. (*r*=−0.447 *p*=0.004, *r*=−0.376 *p*=0.017, *r*=−323 *p*=0.048, and *r*=0.568 *p*=0.01, respectively). There was also a correlation between the increase in TING between the study visits and BRS at follow-up in children with type 1 diabetes (*r*=0.524 *p*=0.031).

**Conclusions/interpretation:**

In this well-regulated type 1 diabetes cohort we did not find manifest signs of CAN in children with type 1 diabetes. These are promising findings and should motivate further to keep striving for normoglycaemia in paediatric diabetes care. Children with both type 1 diabetes and overweight seem more susceptible to early development of CAN and might benefit from earlier and more intensive preventive targeting.

**Graphical Abstract:**

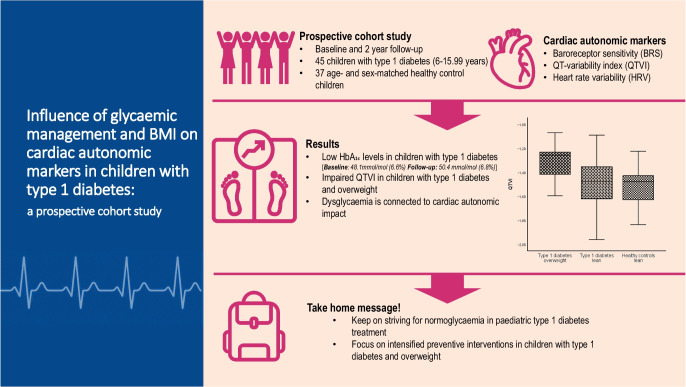

**Supplementary Information:**

The online version contains peer-reviewed but unedited supplementary material available at 10.1007/s00125-025-06592-3.


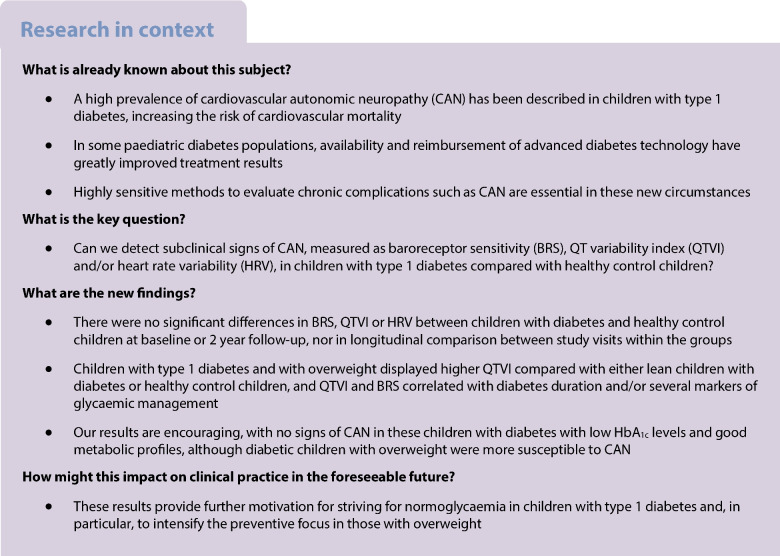


## Introduction

Diabetes autonomic neuropathy (DAN) includes disturbances from the urogenital and gastrointestinal organs, the sudomotor function and cardiovascular autonomic neuropathy (CAN), the latter of which is the most studied form [1]. CAN symptoms include tachycardia, orthostatism and exercise intolerance, and CAN is a known risk factor for cardiovascular mortality in type 1 diabetes [2]. The presence of CAN is also connected with hypoglycaemia unawareness and severe hypoglycaemic events in individuals with type 1 diabetes [3].

CAN prevalence in children with type 1 diabetes varies substantially between different studies, possibly explained by the lack of a gold-standard evaluation method [4, 5]. The SEARCH for diabetes in youth study showed a 14.4% prevalence in young individuals with type 1 diabetes with a mean duration of illness of 7.9 years, measured as impaired heart rate variability (HRV) [6], and the DCCT/EDIC study found a CAN prevalence of 8.5% 6 years after diagnosis, measured as prolonged RR interval [7]. CAN in children with diabetes has previously been associated with type 1 diabetes duration, glycaemic management [5] and a number of traditional cardiovascular risk factors such as hypertension and obesity. It is even more pronounced in young individuals with type 2 diabetes and also in the case of lower HbA_1c_ levels and shorter duration of illness [8]. Although data on the prevalence of CAN in children with type 1 diabetes are inconclusive, studies in adults with type 1 diabetes generally show a high prevalence of CAN (>30%) [9–11] associated with type 1 diabetes duration, hypertension and diabetic kidney disease (DKD) [9, 10, 12]. Hydroxy fatty acids and the tricarboxylic acid cycle are also associated with the development of CAN and may constitute good targets for additional preventive treatment in the future [13].

Hence, trustworthy markers associated with development of CAN in children with type 1 diabetes are essential to enable early detection and treatment before development of manifest CAN [12]. We identify a need for reliable and sensitive methods for early detection of CAN development in children with diabetes, both for providing prevalence numbers to help pathophysiological understanding and for enabling early preventive treatment of CAN in children with diabetes.

### Objectives

Our objectives were to examine the presence of subclinical CAN in children with well-regulated type 1 diabetes compared with age- and sex-matched healthy control children, by measuring baroreceptor sensitivity (BRS), HRV and QT variability index (QTVI), in a cross-sectional case–control study with a 2 year follow-up. First, the presence of impaired cardiac autonomic regulation was evaluated and, second, the used markers for cardiac autonomic regulation were examined in relation to the collected glycaemic and metabolic variables. We hypothesised that detectable impairment of BRS, QTVI and/or HRV can be detected in this study cohort. This impairment is negatively affected by suboptimal glycaemic management and/or metabolic control.

## Methods

### Study design and population

**Fig. 1:**
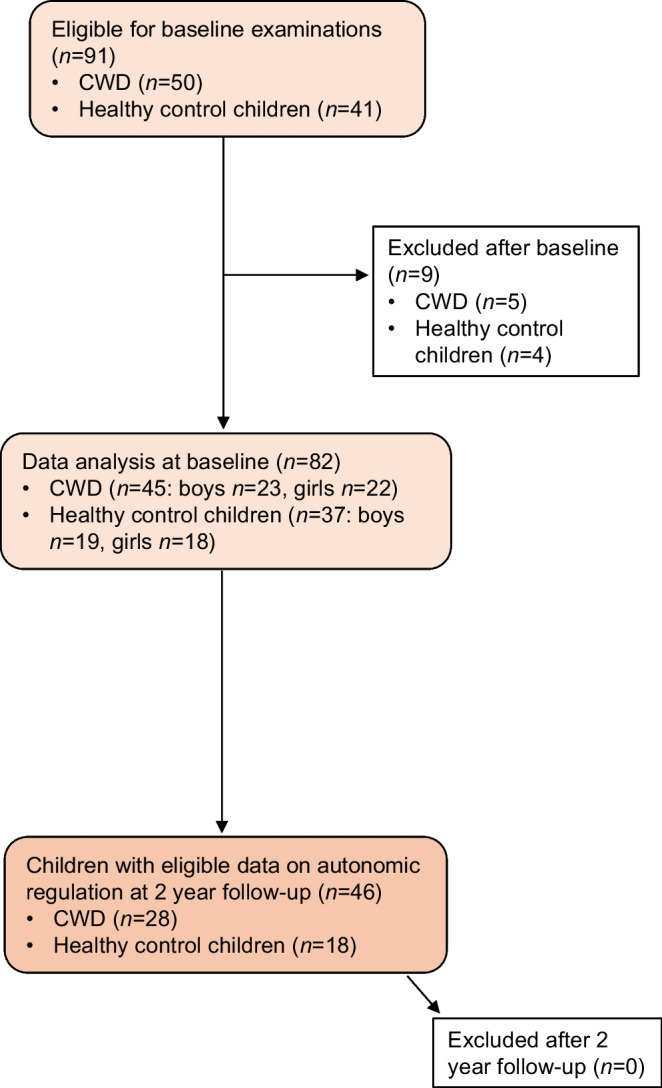
Flow chart showing inclusion and exclusion of study participants during the study course. CWD, children with type 1 diabetes

A prospective cohort study was performed, including children with type 1 diabetes currently monitored at the paediatric diabetology outpatient clinic, at the Queen Silvia Children’s Hospital, Gothenburg, Sweden and age- and sex-matched healthy controls. This is one of Sweden’s largest paediatric diabetes clinics with approximately 650 admitted children with type 1 diabetes. The results are presented according to the STROBE guidelines for cohort studies. Baseline study inclusion was carried out from 25 February 2019 to 28 June 2022, and 2 year follow-up from 26 April 2021 to 8 March 2022. Children with diabetes were asked to participate by the paediatric diabetologist or nurse at a regular outpatient clinic appointment. Healthy control children constituted siblings and friends of the diabetic children, as well as volunteers eligible after announcement in the local area and on social media. An equal proportion of children from each age 6–15.99 years was aimed at in recruitment of the healthy control children. Inclusion criteria for children with diabetes were: age 6–15.99 years; and type 1 diabetes diagnosis ≥5 years ago. Exclusion criteria were other medical treatment and/or condition than type 1 diabetes and insulin; manifest complications such as hypertension and/or dyslipidaemia; pathological findings at baseline examination; or inability to cope with the extensive examination protocol. Fifty children with diabetes and 41 healthy control children were included in the study. Of these, five children with diabetes and four healthy control children were excluded according to some of the exclusion criteria, leaving a total of 45 children with diabetes (boys *n*=23, girls *n*=22) and 37 healthy control children (boys *n*=19, girls *n*=18) included in baseline data analysis. At 2 year follow-up, 28 children with diabetes and 18 healthy control children were included in the final analysis; none were excluded after follow-up examination (Fig. 1). Sex was determined based on the child’s social security number, which is registered at birth. Follow-up was discontinued before all eligible participants had been examined, due to technical failure of the BRS equipment. Participation was voluntary and written consent was collected from all study participants and their caregivers. The study was approved by the local ethics board, Gothenburg 2018-10-12 (Dnr: 622–18) and was conducted according to the Declaration of Helsinki.

Spontaneous BRS, QTVI and HRV were measured using continuous breathing, ECG and BP recordings at rest for 20 min. Blood samples were collected from all study participants at the study visit and urine samples from the same morning were collected. From the Swedish National Diabetes Registry (NDR), HbA_1c_ data from type 1 diabetes diagnosis were available. These data were collected starting from 3 months after type 1 diabetes diagnosis until the last HbA_1c_ value before baseline examination. Mean values and AUCs were calculated and used in data analysis (HbA_1clongitudinal_, HbA_1cAUC_). Continuous glucose monitoring (CGM) data were available in 31 children with diabetes at baseline and 21 at 2 year follow-up. Data from the last 14 consecutive days before the study visit were collected in those who had sensor data for ≥70% of the time [14].

### Spontaneous BRS

Measurement of BRS is a validated non-invasive method for assessment of cardiovascular autonomic regulation in children [15].

Examinations were performed with the participant in a supine position in a quiet and calm surrounding. Continuous BP, three-lead ECG and breathing were recorded for 20 min. ECG recorded the R-wave where the amplitude was at its largest (V5). Finger arterial BP was continuously measured using a plethysmography cuff on the right index finger (Finapres 2300, Ohmeda). The R–R time interval and the number of recorded R-waves were calculated. A baroreflex sequence constituted a paired sequence of increasing BP and RR intervals for three consecutive heartbeats. A built-in algorithm calculated the linear regression slope from three consecutive beats, and a mean linear regression slope (BRS slope) was then calculated from all baroreflex sequences obtained from the 20 min recordings, and considered the BRS [15].

### QTVI

QTVI was calculated from a 5 min recording of the continuous ECG, with <5% occurrence of ectopic heart beats. The examiner defined a QT interval template from the start of the QRS complex to the end of the T-wave, with all possible parts of repolarisation included. The QT template was then used by the built-in algorithm to detect the QT interval from the rest of the beats by stretching or compressing them to match the QT template defined by the examiner. The mean RR interval and variance as well as the mean QT interval and variance is calculated as well as QTVI. QTVI is a logarithmic ratio between normalised QT interval and HRV and was calculated as follows:

$$QTVI={\text{log}}_{10}\left[\frac{\left(\frac{QTv}{QT{m}^{2}}\right)}{\left(\frac{RRv}{RR{m}^{2}}\right)}\right]$$

A one-unit difference in QTVI between two individuals represents a tenfold difference in QT variability [16].

### HRV

HRV was measured during the same ECG recording as QTVI, as described above. Mean RR interval and RR variance were calculated by the built-in algorithm. HRV SDNN is the value presented in the article, which constitutes the SD from all normal RR intervals [17].

### Biochemical analysis

Non-fasting venous blood samples were collected from all study participants after 1–2 h of local anaesthesia. All blood samples were analysed at an accredited laboratory photometrically (Cobas 6000; Roche Diagnostica Scandinavia). C-reactive protein (CRP), white blood cell count (WBC), HbA_1c_, cystatin C and blood lipid levels (total cholesterol, HDL-cholesterol, LDL-cholesterol and triglycerides) were collected. CRP, WBC, HbA_1c_ and cystatin C were analysed with an enzymatic method and serum lipid levels with an enzymatic calorimetric method.

### Statistical analysis

IBM SPSS 23.0 Version 30.0.0.0(172) was used for all statistical analysis, variables were tested for normal distribution by using the Shapiro–Wilk test of normality, and values are presented as mean ± SD or median (range) if not normally distributed. BRS and QTVI *z* scores were calculated using a larger group of healthy control participants from a previous Swedish study comparing cardiac autonomic regulation in children with obesity vs healthy children [18]. Independent sample *t* test was used for comparing children with diabetes and healthy control children, and non-parametric Mann–Whitney *U* test for comparing medians, when the amount of data was too small or the data were not normally distributed. Paired sample *t* test was used in longitudinal analysis, and correlations were analysed using Pearson’s rho, presenting the *r* and *p* values. The sample size was arrived at using the primary outcome measures from this study: measures of vascular wall thickness in the dorsal pedal, radial and carotid arteries. These results have been recently published [19]. For missing data, a variable-specific approach was used, where participants were only excluded from the data analysis including that specific variable. Backwards multivariable regression analysis was performed using important variables found in correlation analysis together with previously known important variables affecting the microvasculature. Values presented are β coefficients, SE, intercept, *R*, *R*^*2*^ values and *p* values.

## Results

### Demographic data

Children with type 1 diabetes and healthy control children were equal in age, height, weight and BMI *z* score (BMIz) at baseline and 2 year follow-up (Table 1). All children with diabetes used a CGM sensor, five at baseline and two at 2 year follow-up were treated with a multiple injection regimen, and 45 at baseline and 26 at follow-up had continuous insulin infusion using an insulin pump. BMIz was ≥1 in 10 and 12 children with diabetes, and 9 and 8 healthy control children at baseline and 2 year follow-up, respectively. None of the children had BMIz ≥2. The mean HbA_1c_ in children with diabetes was 48.1 mmol/mol (6.6%) at baseline and 50.4 mmol/mol (6.8%) at 2 year follow-up (Table 1). Mean time in range (TIR) (3.9–10.0 mmol/l [70–180 mg/dl] glucose) was 64.4% and 68.1% in children with diabetes at baseline and 2 year follow-up, respectively; mean time in normoglycaemia (TING) (3.9–7.8 mmol/l [70–140 mg/dl]) was 42.1 and 48.0% in children with diabetes at baseline and 2 year follow-up, respectively. TING was significantly higher at 2 year follow-up compared with baseline (*p*=0.042). Boys spent more time below range (<3.9 mmol/l glucose) (*p*=0.006) and had a higher CV (*p*=0.012) than girls at baseline (Table 2).

**Table 1:** Anthropometric data at baseline and 2 year follow-up in children with type 1 diabetes vs healthy control children

Characteristic	Baseline			2 year follow-up		
	CWD (*n*=45)	HC (*n*=37)	*p* value	CWD (*n*=28)	HC (*n*=19)	*p* value
Age (years)	12.0 ± 2.3	11.3 ± 2.4	0.192	14.1 ± 2.3	13.1 ± 3.1	0.197
Type 1 diabetes duration (years)	7.7 ± 1.8	–	–	9.6 ± 1.7	–	–
Time to follow-up (years)	–	–	–	2.1 ± 0.14	2.1 ± 0.21	0.679
Height (cm)	156.5 ± 15.0	152.3 ± 16.3	0.228	166.0 ± 12.6	160.1 ± 18.7	0.198
Weight (kg)	48.6 ± 14.4	44.7 ± 13.7	0.222	60.2 ± 14.6	53.4 ± 17.0	0.143
Waist circumference (cm)	69.2 ± 7.4	67.9 ± 9.3	0.467	73.2 ± 9.4	69.4 ± 8.0	0.163
Hip circumference (cm)	85.2 ± 9.5	82.2 ± 11.0	0.199	94.4 ± 11.2	89.1 ± 11.8	0.129
BMIz	0.4 ± 0.8	0.3 ± 0.9	0.731	−0.1 ± 0.7	0.1 ± 0.7	0.823
SBP (mmHg)	106 ± 7	103 ± 8	0.120	112 ± 9	108 ± 10	0.137
DBP (mmHg)	66 ± 4	63 ± 5	0.005	69 ± 5	63 ± 5	< 0.001
Office SBPz	0.5 ± 0.2	0.5 ± 0.2	0.378	0.6 ± 0.2	0.5 ± 0.2	0.256
Office DBPz	0.6 ± 0.2	0.5 ± 0.2	0.019	0.6 ± 0.2	0.5 ± 0.2	0.005
Blood samples						
HbA_1c_ (mmol/mol)	48.1 ± 6.0	31.1 ± 2.22	< 0.01	50.4 ± 7.0	31.0 ± 3.5	< 0.001
HbA_1c_ (%)	6.6 ± 0.6	5.0 ± 0.2	< 0.01	6.8 ± 0.6	5.0 ± 0.3	< 0.001
Cystatin C (mg/l)	0.9 ± 0.1	0.9 ± 0.1	0.259	1.0 ± 0.8	0.8 ± 0.1	0.454
eGFR (ml/min per 1.73m^2^)	104.3 ± 16.9	111.2 ± 16.4	0.080	109.8 ± 16.2	112.3 ± 20.3	0.690
Urine albumin/creatinine (mg/mmol)	0.8 ± 0.9	0.8 ± 0.4	0.944	1.7 ± 2.1	0.7 ± 0.4	0.090
Cholesterol (mmol/l)	4.1 ± 0.6	4.0 ± 0.6	0.290	3.9 ± 0.8	3.9 ± 0.7	0.921
Triglycerides (mmol/l)	0.7 ± 0.6	1.1 ± 0.5	<0.01	0.8 ± 0.3	1.1 ± 0.5	0.041
HDL (mmol/l)	1.5 ± 0.2	1.3 ± 0.3	<0.01	1.7 ± 0.9	1.4 ± 0.2	0.132
LDL (mmol/l)	2.3 ± 0.5	2.5 ± 0.6	0.308	2.4 ± 0.6	2.4 ± 0.6	0.948

Values are presented as mean ± SD

Comparison between groups was with independent sample *t* test

CWD, children with type 1 diabetes; DBP, diastolic BP; HC, healthy control children; SBP, systolic BP

**Table 2:** Glycaemic control baseline and follow-up. Baseline comparison between boys and girls with type 1 diabetes

Variable	Longitudinal comparison			Comparison of boys and girls at baseline		
	CWD baseline (*n*=45)	CWD 2-year follow-up (*n*=25)	Longitudinal *p* value	Baseline boys (*n*=23)	Baseline girls (*n*=22)	*p* value
TIR (%)	64.4 ± 8.8	68.1 ± 12.6	0.201	64.4 ± 8.8	64.4 ± 9.1	0.705
TING (%)	42.1 ± 9.4	48.0 ± 10.9	0.042	43.6 ± 8.0	40.4 ± 10.8	0.586
TBR (%)	3.9 ± 2.7	5.8 ± 3.9	0.061	5.1 ± 2.8	2.6 ± 1.9	0.006
Mean glucose (mmol/l)	8.6 ± 0.99	8.2 ± 1.13	0.165	8.3 ± 1.0	8.9 ± 1.0	0.250
CV (%)	39.6 ± 5.0	39.1 ± 6.0	0.883	41.0 ± 4.6	37.4 ± 4.6	0.012
Longitudinal HbA_1c_ (mmol/mol)	51.4 ± 6.6	–	–	51.8 ± 6.3	51.0 ± 6.9	
Longitudinal HbA_1c_ (%)	6.9 ± 2.8	–	–	6.9 ± 2.7	6.9 ± 2.8	0.547
HbA_1cAUC_	372.58 ± 121.27	–	–	383.59±115.52	361.07±128.70	0.540

Data are presented as mean ± SD

Comparison between groups with independent sample *t* test and paired sample *t* test for longitudinal analysis

CWD, children with type 1 diabetes; TBR, time below range (<3.9 mmol/l glucose); TING, time in normoglycaemia (3.9–7.8 mmol/l glucose); TIR, time in range (3.9–10.0 mmol/l glucose)

SD=SD from mean glucose mmol/l

As previously reported [19], diastolic BP *z* score (DBPz) was significantly higher in children with diabetes than in healthy control children at baseline and 2 year follow-up (*p*=0.019 and *p*=0.005, respectively) (Table 1). Baseline office diastolic BP was higher in children with diabetes irrespective of sex (girls *p*=0.043, boys *p*=0.054) (electronic supplementary material [ESM] Table 1). Children with diabetes showed lower triglycerides at baseline and 2 year follow-up (*p*<0.01 and *p*=0.041, respectively), and higher HDL at baseline (*p*<0.01) compared with healthy control children (Table 1).

### Autonomic regulation of the heart

No differences in BRS, QTVI or HRV were found between children with diabetes and healthy control children at baseline or 2 year follow-up, neither in longitudinal comparison between baseline and follow-up or in separate analysis with groups stratified by sex. No longitudinal change was found in any of the autonomic variables in children with or without diabetes (Table 3 and ESM Table 2).

**Table 3:** Cardiac autonomic regulation in children with type 1 diabetes compared with healthy control children at baseline and at 2 year follow-up, with longitudinal analysis

BRS	Baseline^a^			2 year follow-up			Longitudinal *p* value CWD, HC
	CWD (*n*=38)	HC (*n*=32)	*p* value	CWD (*n*=28)	HC (*n*=18)	*p* value	
BRS slope (ms/mmHg)	19.1 ± 7.8	19.5 ± 7.1	0.831	19.0 ± 7.2	21.5 ± 6.9	0.345	0.780, 0.098
BRS *z* score	−0.88 ± 0.78	−0.05 ± 0.71	0.831	−0.12 ± 0.70	0.08 ± 0.67	0.345	0.780, 0.098
QTVI	−1.46 ± 0.24	−1.48 ± 0.26	0.712	−1.35 ± 0.24	−1.53 ± 0.26	0.060	0.178, 0.601
QTVI *z* score	0.12 ± 0.24	0.05 ± 0.78	0.712	0.35 ± 0.25	−0.09 ± 0.76	0.060	0.174, 0.601
HRV SDNN (ms)	81.2 ± 32.9	91.8 ± 35.8	0.185	76.8 ± 24.7	91.8 ± 30.9	0.121	0.865, 0.909

Data are presented as mean ± SD

^a^*n*<45 for CWD and *n*<37 for HC at baseline because of either technical issues with the BRS equipment or poor quality of the examination, e.g. if a child was unable to be sufficiently still throughout the examination

Comparison between groups with independent sample *t* test and paired sample *t* test for longitudinal analysis

CWD, children with type 1 diabetes; HC, healthy control children; SDNN, SD from all normal RR intervals

Children with diabetes with BMIz ≥1 at baseline (*n*=9) had a higher QTVI than either diabetic children with BMIz <1 or healthy control children with normal weight (median [range] QTVI: for children with diabetes BMIz ≥1, −1.26 [−1.69 to 1.07]; for children with diabetes BMIz <1, −1.48 [−2.08 to −1.09]; and for healthy control children, −1.47 [−1.85 to −0.55]; *p*=0.031 and *p*=0.024, respectively).

### Correlations

**Fig. 2:**
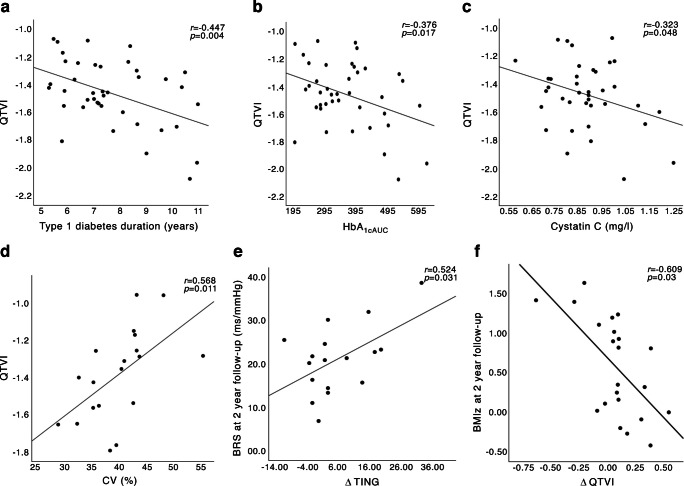
Correlations between autonomic measures and glycaemic and metabolic variables in children with type 1 diabetes. Correlation analysis using Pearson’s rho, *r* and *p* values are presented in each scatter plot. Correlations are shown for QTVI with type 1 diabetes duration (**a**), QTVI with HbA_1cAUC_ (**b**), QTVI with cystatin C (**c**), QTVI with CV (%) (**d**), BRS at 2 year follow-up (ms/mmHg) with ΔTING (**e**), and BMIz at 2 year follow-up with $\Delta$ QTVI (**f**)

Correlations between BRS slope, QTVI and HRV were found in both children with type 1 diabetes and healthy control children (*r*=−0.392–0.733, *p*<0.001–0.035). At baseline in children with diabetes, QTVI was negatively correlated with type 1 diabetes duration, HbA_1cAUC,_ and cystatin C (*r*=−0.447 *p*=0.004, *r*=–0.376 *p*=0.017, and *r*=–323 *p*=0.048, respectively) (Fig. 2). Cystatin C was positively correlated with HbA_1cAUC,_ CV, type 1 diabetes duration and age (HbA_1cAUC_, *r*=0.404, *p*<0.01; CV, *r*=0.381, *p*=0.041; type 1 diabetes duration, *r*=0.342, *p*=0.025; age, *r*=0.397 *p*=0.008) and negatively correlated with TIR (*r*=–0.476, *p*=0.009).

In healthy control children, HRV was negatively correlated with SBP *z* score (SBPz) (*r*=–0.413, *p*=0.015) and DBPz (*r*=−0.433, *p*=0.010) and positively correlated with HDL (*r*=0.389, *p*=0.031).

At 2 year follow-up, BRS slope correlated positively with the difference in TING between baseline and follow-up (ΔTING) (*r*=0.524, *p*=0.031) in children with diabetes (Fig. 2). There were also positive correlations between QTVI and CV, and between cystatin C and BMIz (*r*=0.568, *p*=0.011; and *r*=0.422, *p*=0.028, respectively), and a negative correlation between the change in QTVI from baseline to follow-up (ΔQTVI) and BMIz (*r*=–0.609, *p*=0.03) (Fig. 2).

In healthy control children, QTVI correlated with difference in BMIz between baseline and follow-up (ΔBMIz), DBPz and sex at 2 year follow-up (*r*=0.617, *p*=0.008; *r*=0.583, *p*=0.011; and *r*=0.483, *p*=0.042, respectively).

### Regression analysis

In univariate regression, QTVI was associated with type 1 diabetes duration and cystatin C. Backwards multivariable regression in children with diabetes, including BRS slope, QTVI and HRV as dependent variables and type 1 diabetes duration, sex, BMIz, SBPz and DBPz, HbA_1c_, CV and cystatin C as independent variables, resulted in a significant model for QTVI including age, type 1 diabetes duration, HbA_1c_ and cystatin C (*R*=0.693, *p*=0.0002), with each independent variable separately significant (ESM Table 3). The same model was used for HRV, resulting in one significant model including HbA_1c_ and cystatin C, where HbA_1c_ was separately significant (*R*=0.390, *p*=0.047). In backwards multivariable regression on total study population, age, sex, type 1 diabetes diagnosis, BMIz and BP *z* scores, HbA_1c_ and cystatin C were used as independent variables. This analysis resulted in several significant models for QTVI, the first of which included all independent variables (*R*=0.477, *p*=0.050) and the last model included age, SBPz and cystatin C as independent variables (*R*=0.391, *p*=0.016). Significant models for HRV, where the first one included age, sex, HbA_1c_, BP *z* scores and cystatin C as independent variables (*R*=0.432, *p*=0.0369) and the last only DBPz (*R*=0.334, *p*=0.0047) were also found (ESM Table 3).

## Discussion

In this prospective cohort study, we used highly sensitive methods for detection of subclinical CAN in children with type 1 diabetes with low HbA_1c_ and no presence of other traditional cardiovascular risk factors. Children with type 1 diabetes were compared with age- and sex-matched healthy control children, and we found no differences in BRS, QTVI or HRV between the study groups at baseline or at 2 year follow-up, nor any longitudinal changes in either of the study groups. There was an improvement in TING in children with diabetes from baseline to follow-up, correlating with higher BRS at follow-up, and QTVI was higher in children with diabetes with BMIz ≥1 compared with children both with and without diabetes and with normal weight. QTVI correlated with type 1 diabetes duration and HbA_1cAUC_ at baseline and type 1 diabetes duration, cystatin C and age were independent determinants for QTVI in children with diabetes in multivariable regression. Our results, especially the correlations found for QTVI and BRS, as well as the results from backwards multivariable regression suggest an association between dysglycaemia and CAN, even in this study cohort where children with diabetes display comparably low HbA_1c_ levels, even better lipid profiles than matched healthy control children, and no other manifest cardiovascular complications. These quite unique glycaemic and metabolic management results possibly explain the findings that there were no significant differences in any of the measures of subclinical CAN between children with and without diabetes.

Our results are in line with several previous studies, showing an increased prevalence of DAN in children with diabetes, mostly measured as decreased HRV and/or impaired cardiovascular reflex test (CVRT) [20–23]. One study showed decreased HRV, presence of postural hypotension and diastolic ventricular dysfunction in 30 children with diabetes (mean ± SD diabetes duration 8 ± 3.66 years) and HbA_1c_ 100 ± 23 mmol/mol (11.34 ± 2.1%) [21]. Decreased BRS was found in a cohort of adolescents with type 1 diabetes, connected to HbA_1c_, type 1 diabetes duration and BP [24]. Another study used several bedside and confirmatory methods for examining large- and small-fibre neuropathy in adolescents with type 1 diabetes where at least 40% had one pathological autonomic test. The highest prevalence of autonomic neuropathy was seen in the group of adolescents featuring the highest longitudinal HbA_1c_ levels, and total insulin dose, age, triglyceride levels and previous smoking increased the relative risk of developing autonomic neuropathy [22]. In a recent Danish study, the prevalence of early CAN was 17% and the prevalence of manifest CAN was 3% in a cohort of 60 adolescents with type 1 diabetes compared with 23 healthy control counterparts [23]. In that study, median (IQR) type 1 diabetes duration was 4.8 (2.7–7.7) years and HbA_1c_ was 54 (49–63) mmol/mol (7.1 [6.6–7.9]%), and no significant correlating markers for CAN, such as higher HbA_1c_ or longer type 1 diabetes duration, were identified. However, the DCCT/EDIC study found that the most important risk factors for CAN were older age, higher mean HbA_1c_, sustained albuminuria and type 1 diabetes duration [9]. The DCCT/EDIC study population was older at inclusion (median [range] 27 [22, 32] years) and with a higher HbA_1c_ (median [range] 72.7 [62.2, 86.7] mmol/mol; 8.8 [7.8, 10.1]%) than the population in the previously described Danish study and compared with children with diabetes in our study cohort. Hence, we consider our findings in line with these earlier studies, showing HbA_1c_ levels higher than for the children with diabetes in our study cohort, possibly explaining detectable autonomic dysfunction in children with diabetes [21, 23–25]. Connections between glucometrics and CAN have been observed in adults with type 1 diabetes before [26–28], which is also in line with the correlations found in this study between QTVI and BRS and a few of the glucometrics. Our findings are encouraging, implying that good glycaemic management can contribute to delaying or preferably preventing the development of CAN, as previously has been shown [29].

Interestingly, QTVI seemed to be the most affected measure of CAN in this study cohort. QTVI is likely to reflect sympathetic and vagal nerve balance in the cardiac cycle [30], and Berger et al found that increased QTVI in patients with heart failure meant a higher risk of severe arrythmias [16], possibly indicating a higher risk in case of hypoglycaemia. A previous study in adults with type 2 diabetes and manifest CAN compared the feasibility of HRV and QTVI in assessment of CAN progression and showed that QTVI was more sensitive in discriminating between the early and later stages of CAN and therefore considered useful in evaluating CAN progression in individuals with diabetes [31].

Obesity in childhood is in itself a risk factor for CAN [18], and microvascular complications are more frequent in children with type 2 diabetes, who more often display several traditional cardiovascular risk factors such as obesity and/or hypertension in addition to their diabetes diagnosis [6, 32]. Due to these earlier findings, we decided to stratify our study groups according to BMIz. Analysing the subgroup of children with diabetes with BMIz ≥1, we found elevated QTVI compared with either children with diabetes without overweight or healthy control children with normal weight. This is also in line with previous studies where BMI has been associated with the presence of CAN in diabetes [8, 12, 33], and implies an increased susceptibility to autonomic dysfunction in children with diabetes with overweight, suggesting a need for intensified cardiovascular preventive actions in this subgroup.

The connection found between cystatin C and QTVI is also consistent with previous studies wherein CAN has been associated with increased albuminuria and decreased GFR in both adolescents and adults with type 1 diabetes [8, 9, 11]. Our findings further suggest an association between microvascular impact and glycaemic management, with the correlations between cystatin C and glucometrics.

Strengths of the study include the multiple methods used for assessment of CAN and the continuity of staff throughout the course of the study. Another strength is the prospective study design and the well-described cohort of children with type 1 diabetes, displaying excellent glycaemic management, and the age- and sex-matched healthy control group. The study population is considered representative for children with type 1 diabetes in Sweden, where 42% of the children presented with HbA_1c_ ≤48mmol/mol (6.5%) during the last 12 months [34], as well as other similar paediatric type 1 diabetes populations with highly available advanced technology. Due to national regulations, information about ethnicity was not possible to include in this study. However, we consider information about ethnic background to be of limited importance due to the smaller scale of the study. Information about sex was based on the child’s social security number, which is registered at birth. In this study only the biological vascular differences were considered, which is why this was the preferable method. The healthy control children were recruited mainly through the children with diabetes (siblings/ friends) and the children are likely to come from similar socioeconomic backgrounds. However, in-depth information about socioeconomic life circumstances was not collected.

The relatively small number of study participants is an important limitation to the study, as well as the limited number of children with BMIz ≥1. The prospective study design and the matched healthy control group somewhat compensate for this limitation, and in data analysis comparing children with diabetes and with overweight vs children with diabetes and healthy control children with normal weight, non-parametric Mann–Whitney *U* tests were used. Another limitation is that inclusion took place during the pandemic. This prolonged the time for study inclusion and meant that some of our study participants were exposed to the COVID-19 virus before, and some after, their participation. There was no possibility of excluding individuals who had the infection before baseline. The introduction of advanced insulin delivery by hybrid closed-loop systems also took place during this time. Hence, diabetes treatment might differ between the children with diabetes included at the beginning of the study compared with those who were included later.

To summarise, even in this cohort of children with type 1 diabetes, with what is considered good glycaemic and metabolic management, we find a connection between dysglycaemia and CAN. We also find an impairment of QTVI in children with diabetes with BMIz ≥1, confirming the increased risk of autonomic dysfunction associated with overweight/obesity. QTVI seems the more sensitive marker for CAN, possibly mirroring both parasympathetic and sympathetic regulation of the heart.

For paediatric type 1 diabetes populations, such as in Sweden, where advanced diabetes care is reimbursed and available, rapid improvement of treatment results have occurred. Similar studies, with larger cohorts, will be essential to increase understanding of CAN development in these new and better circumstances for treatment of children with diabetes. Hence, our results could serve as motivation to keep improving glycaemic management, even in the era of access to high technology, to even further reduce the risk of chronic complications such as CAN. Our results also suggest that intensified focus on cardiovascular prevention would be beneficial in children with type 1 diabetes and with overweight/obesity.

### Conclusion

In type 1 diabetes populations with access to high technology and improved glycaemic management, data on chronic complication development are still limited, and our study adds to this knowledge gap. Encouragingly, we find no subclinical signs of CAN in this cohort of children with well-regulated type 1 diabetes with normal weight. Hence, sustaining low HbA_1c_ and high TIR and TING might delay or preferably prevent development of CAN. We further confirm the additional risk of chronic complications that comes with overweight/obesity in type 1 diabetes, suggesting that intensified cardiovascular preventive focus in this subgroup might be beneficial. Further studies with larger study cohorts are essential to confirm our results and to continue increasing the knowledge behind cardiovascular complication development in children with well-regulated type 1 diabetes. Hopefully, this will result in even further improvement of cardiovascular prevention in children with diabetes, increasing both life expectancy and quality of life for people living with type 1 diabetes in the future.

## Supplementary Information

Below is the link to the electronic supplementary material.

ESM Tables (PDF 294 KB)

## Data Availability

The data that support these study findings are stored in a controlled database at the Sahlgrenska University Hospital, Gothenburg, Sweden. Data are not openly accessible but are available upon request to the corresponding author.

## Abbreviations

BMIz: BMI *z* score
BRS: Baroreceptor sensitivity
CAN: Cardiovascular autonomic neuropathy
CGM: Continuous glucose monitoring
CRP: C-reactive protein
DAN: Diabetes autonomic neuropathy
DBPz: Diastolic BP *z* score
HRV: Heart rate variability
QTVI: QT variability index
SBPz: Systolic BP *z* score
TING: Time in normoglycaemia
TIR: Time in range
WBC: White blood cell count

## References

[1] Mizokami-Stout K, Bailey R, Ang L et al (2022) Symptomatic diabetic autonomic neuropathy in type 1 diabetes (T1D): findings from the T1D exchange. J Diabetes Complicat 36(5):108148. 10.1016/j.jdiacomp.2022.108148 10.1016/j.jdiacomp.2022.108148 35279403

[2] Spallone V, Ziegler D, Freeman R et al (2011) Cardiovascular autonomic neuropathy in diabetes: clinical impact, assessment, diagnosis, and management. Diabetes Metab Res Rev 27(7):639–53. 10.1002/dmrr.1239 21695768 10.1002/dmrr.1239

[3] da Silva TPB, Rolim LC, de Camargo Sallum Filho CF, Zimmermann LM, Malerbi F, Dib SA (2015) Impaired awareness of hypoglycemia is associated with progressive loss of heart rate variability in patients with type 1 diabetes. Diabetol Metab Syndr 7(1):63. 10.1186/1758-5996-7-S1-A6326288659

[4] Rasmussen VF, Jensen TS, Tankisi H et al (2021) Large fibre, small fibre and autonomic neuropathy in adolescents with type 1 diabetes: a systematic review. J Diabetes Complicat 35(11):108027. 10.1016/j.jdiacomp.2021.108027 10.1016/j.jdiacomp.2021.108027 34429229

[5] Tang M, Donaghue KC, Cho YH, Craig ME (2013) Autonomic neuropathy in young people with type 1 diabetes: a systematic review. Pediatr Diabetes 14(4):239–48. 10.1111/pedi.12039 23627912 10.1111/pedi.12039

[6] Dabelea D, Stafford JM, Mayer-Davis EJ et al (2017) Association of type 1 diabetes vs type 2 diabetes diagnosed during childhood and adolescence with complications during teenage years and young adulthood. JAMA 317(8):825–35. 10.1001/jama.2017.0686 28245334 10.1001/jama.2017.0686 PMC5483855

[7] (1998) The effect of intensive diabetes therapy on measures of autonomic nervous system function in the Diabetes Control and Complications Trial (DCCT). Diabetologia 41(4):416-23. 10.1007/s001250050924 10.1007/s001250050924 PMC2635092 9562345

[8] Varley BJ, Gow ML, Cho YH et al (2022) Higher frequency of cardiovascular autonomic neuropathy in youth with type 2 compared to type 1 diabetes: role of cardiometabolic risk factors. Pediatr Diabetes 23(7):1073–9 10.1111/pedi.13393 PMC9805172 35856852

[9] Braffett BH, Gubitosi-Klug RA, Albers JW et al (2020) Risk factors for diabetic peripheral neuropathy and cardiovascular autonomic neuropathy in the Diabetes Control and Complications Trial/Epidemiology of Diabetes Interventions and Complications (DCCT/EDIC) Study. Diabetes 69(5):1000–10. 10.2337/db19-1046 32051148 10.2337/db19-1046 PMC7171957

[10] Tannus LR, Drummond KR, Clemente EL, da Matta Mde F, Gomes MB (2014) Predictors of cardiovascular autonomic neuropathy in patients with type 1 diabetes. Front Endocrinol (Lausanne) 5:191. 10.3389/fendo.2014.00191 25505446 10.3389/fendo.2014.00191 PMC4243695

[11] Bjerre-Christensen T, Winther SA, Tofte N et al (2021) Cardiovascular autonomic neuropathy and the impact on progression of diabetic kidney disease in type 1 diabetes. BMJ Open Diabetes Res Care 9(1):e002289. 10.1136/bmjdrc-2021-002289 34645614 10.1136/bmjdrc-2021-002289 PMC8515448

[12] Serdarova M, Dimova R, Chakarova N, Grozeva G, Todorova A, Tankova T (2021) Relationship between cardiac autonomic neuropathy and cardio-metabolic risk profile in adults with type 1 diabetes. Diabetes Res Clin Pract 174:108721. 10.1016/j.diabres.2021.108721 33640411 10.1016/j.diabres.2021.108721

[13] Hansen CS, Suvitaival T, Theilade S et al (2022) Cardiovascular autonomic neuropathy in type 1 diabetes is associated with disturbances in TCA, Lipid, and glucose metabolism. Front Endocrinol (Lausanne) 13:831793. 10.3389/fendo.2022.831793 35498422 10.3389/fendo.2022.831793 PMC9046722

[14] Battelino T, Alexander CM, Amiel SA et al (2023) Continuous glucose monitoring and metrics for clinical trials: an international consensus statement. Lancet Diabetes Endocrinol 11(1):42–57. 10.1016/S2213-8587(22)00319-9 36493795 10.1016/S2213-8587(22)00319-9

[15] Parlow J, Viale JP, Annat G, Hughson R, Quintin L (1995) Spontaneous cardiac baroreflex in humans. Comparison with drug-induced responses. Hypertension 25(5):1058–68. 10.1161/01.hyp.25.5.1058 7737717 10.1161/01.hyp.25.5.1058

[16] Berger RD, Kasper EK, Baughman KL, Marban E, Calkins H, Tomaselli GF (1997) Beat-to-beat QT interval variability: novel evidence for repolarization lability in ischemic and nonischemic dilated cardiomyopathy. Circulation 96(5):1557–65. 10.1161/01.cir.96.5.1557 9315547 10.1161/01.cir.96.5.1557

[17] (1996) Heart rate variability: standards of measurement, physiological interpretation and clinical use. Task Force of the European Society of Cardiology and the North American Society of Pacing and Electrophysiology. Circulation 93(5):1043-65 8598068

[18] Dangardt F, Volkmann R, Chen Y, Osika W, Marild S, Friberg P (2011) Reduced cardiac vagal activity in obese children and adolescents. Clin Physiol Funct Imaging 31(2):108–13. 10.1111/j.1475-097X.2010.00985.x 21087396 10.1111/j.1475-097X.2010.00985.x

[19] Bergdahl E, Forsander G, Sundberg F, Milkovic L, Dangardt F (2025) Investigating the presence and detectability of structural peripheral arterial changes in children with well-regulated type 1 diabetes versus healthy controls using ultra-high frequency ultrasound: a single-centre cross-sectional and case-control study. EClinicalMedicine 81:103097. 10.1016/j.eclinm.2025.103097 40034566 10.1016/j.eclinm.2025.103097 PMC11872503

[20] Ewing DJ, Martyn CN, Young RJ, Clarke BF (1985) The value of cardiovascular autonomic function tests: 10 years experience in diabetes. Diabetes Care 8(5):491–8. 10.2337/diacare.8.5.491 4053936 10.2337/diacare.8.5.491

[21] Gözüküçük D, İleri BA, Başkan SK et al (2024) Evaluation of cardiac autonomic dysfunctions in children with type 1 diabetes mellitus. BMC Pediatrics 24(1):229. 10.1186/s12887-024-04644-y 38561716 10.1186/s12887-024-04644-y PMC10986024

[22] Rasmussen VF, Thrysøe M, Nyengaard JR et al (2023) Neuropathy in adolescents with type 1 diabetes: confirmatory diagnostic tests, bedside tests, and risk factors. Diabetes Res Clin Pract 201:110736. 10.1016/j.diabres.2023.110736 37276985 10.1016/j.diabres.2023.110736

[23] Damm JA, Dalgas-Madsen A, Hansen CS et al (2024) Presence of neuropathy in children and adolescents with type 1 diabetes evaluated with bedside modalities. J Diabetes Complicat 38(11):108873. 10.1016/j.jdiacomp.2024.108873 10.1016/j.jdiacomp.2024.108873 39306874

[24] DallaPozza R, Bechtold S, Bonfig W et al (2007) Impaired short-term blood pressure regulation and autonomic dysbalance in children with type 1 diabetes mellitus. Diabetologia 50(12):2417–23. 10.1007/s00125-007-0823-9 17898991 10.1007/s00125-007-0823-9

[25] Velayutham V, Benitez-Aguirre P, Craig M, Cho YH, Liew G, Donaghue K (2022) Cardiac autonomic nerve dysfunction predicts incident retinopathy and early kidney dysfunction in adolescents with type 1 diabetes. Diabetes Care 45(10):2391–5. 10.2337/dc22-0349 35997303 10.2337/dc22-0349

[26] Jun JE, Lee SE, Lee YB et al (2019) Continuous glucose monitoring defined glucose variability is associated with cardiovascular autonomic neuropathy in type 1 diabetes. Diabetes Metab Res Rev 35(2):e3092. 10.1002/dmrr.3092 30345631 10.1002/dmrr.3092

[27] Guo Q, Zang P, Xu S et al (2020) Time in range, as a novel metric of glycemic control, is reversely associated with presence of diabetic cardiovascular autonomic neuropathy independent of HbA1c in Chinese type 2 diabetes. J Diabetes Res 2020:5817074. 10.1155/2020/5817074 32090120 10.1155/2020/5817074 PMC7026737

[28] Nyiraty S, Pesei F, Orosz A et al (2018) Cardiovascular autonomic neuropathy and glucose variability in patients with type 1 diabetes: is there an association? Front Endocrinol (Lausanne) 9:174. 10.3389/fendo.2018.00174 29725320 10.3389/fendo.2018.00174 PMC5916962

[29] Pop-Busui R, Low PA, Waberski BH et al (2009) Effects of prior intensive insulin therapy on cardiac autonomic nervous system function in type 1 diabetes mellitus: the Diabetes Control and Complications Trial/Epidemiology of Diabetes Interventions and Complications study (DCCT/EDIC). Circulation 119(22):2886–93. 10.1161/CIRCULATIONAHA.108.837369 19470886 10.1161/CIRCULATIONAHA.108.837369 PMC2757005

[30] Kusuki H, Tsuchiya Y, Mizutani Y et al (2020) QT variability index is correlated with autonomic nerve activity in healthy children. Pediatr Cardiol 41(7):1432–7. 10.1007/s00246-020-02399-8 32572546 10.1007/s00246-020-02399-8 PMC7557489

[31] Khandoker AH, Imam MH, Couderc JP, Palaniswami M, Jelinek HF (2012) QT variability index changes with severity of cardiovascular autonomic neuropathy. IEEE Trans Inf Technol Biomed 16(5):900–6. 10.1109/TITB.2012.2205010 22929462 10.1109/TITB.2012.2205010

[32] Aulich J, Cho YH, Januszewski AS et al (2019) Associations between circulating inflammatory markers, diabetes type and complications in youth. Pediatr Diabetes 20(8):1118–27. 10.1111/pedi.12913 31464058 10.1111/pedi.12913

[33] Franceschi R, Mozzillo E, Di Candia F et al (2022) A systematic review of the prevalence, risk factors and screening tools for autonomic and diabetic peripheral neuropathy in children, adolescents and young adults with type 1 diabetes. Acta Diabetol 59(3):293–308. 10.1007/s00592-022-01850-x 35089443 10.1007/s00592-022-01850-x

[34] Nationella Diabetesregistret, Knappen. Available from: https://ndr.registercentrum.se/statistik/statistikvisning-knappen-and-profilen/p/ByTsngbCj. Accessed 30 May 2025

