# Supplementary material for: Influence of glycaemic management and BMI on cardiac autonomic markers in children with type 1 diabetes: a prospective cohort study
Source: Diabetologia. 2025 Nov 11;69(2):480–90. doi: 10.1007/s00125-025-06592-3 (PMC12779695; doi:10.1007/s00125-025-06592-3)
Supplement: Supplementary file 1 — ESM Tables (PDF 294 KB) [file 125_2025_6592_MOESM1_ESM.pdf]

ESM table 1: Baseline anthropometric data and blood samples. CWD compared to healthy controls, groups stratified by sex.

ESM table 1. Baseline characteristics. groups stratified by sex						
	Girls CWD n=22	Girls HC n=18	p-value	Boys CWD n=23	Boys HC n=19	p-value
**Age (years)**	11.92 ± 2.36	11.41 ± 2.56	0.509	12.14 ±2.37	11.27±2.44	0.249
**T1D duration (years)**	7.58±2.02	--	--	7.80±1.61	--	--
**Height (cm)**	153.57±14.25	151.27±15.26	0.625	159.34±15.40	153.30±17.67	0.244
**Weight (kg)**	46.58±14.64	43.13±12.01	0.427	50.45±14.23	46.22±15.23	0.359
**Waist**	68.17±8.49	66.56±8.97	0.576	70.17±6.29	69.00±9.69	0.639
**Hip**	84.43±9.81	82.32±10.05	0.519	85.80±9.36	82.05±12.04	0.262
**BMI z-score (SD)**	0.27±0.81	0.14±0.85	0.615	0.42±0.73	0.43±0.92	0.969
**SBP (mmHg)**	104±5	103±7	0.636	108±8	103±9	0.120
**DBP (mmHg)**	66±4	63±5	0.043	65±4	62±6	0.054
**Office SBP z-score**	0.49±0.25	0.45±0.25	0.638	0.50±0.21	0.45±0.21	0.433
**Office DBP z-score**	0.60±0.17	0.53±0.16	0.158	0.58±0.17	0.47±0.19	0.066
**Blood samples**						
**HbA1c (mmol/mol)**	47.61±5.99	31.78±2.02	<0.001	48.57±6.07	30.31±2.24	<0.001
**HbA1c (%)**	6.51±0.55	5.06±0.18	<0.001	6.60±0.56	4.92±0.21	<0.001
**Cystatin C (mg/L)**	0.90±0.15	0.87±0.12	0.339	0.88±0.12	0.84±0.09	0.483
**eGFR (ml/min)**	101.47±16.93	108.94±17.54	0.213	106.95±16.84	113.81±15.20	0.196
**Urine albumin/creatinine (mg/mmol)**	0.66±0.79	0.67±0.29	0.967	1.26±1.24	0.71±0.40	0.112
**Cholesterol (mmol/L)**	4.11±0.70	3.88±0.62	0.529	4.08±0.38	4.18±0.62	0.297
**Triglycerides(mmol/L)**	0.65±0.28	1.05±0.45	0.064	0.79±0.26	1.04±0.52	0.002
**HDL (mmol/L)**	1.54±0.27	1.27±0.26	0.043	1.45±0.17	1.30±0.27	0.003
**LDL (mmol/L)**	2.29±0.64	2.34±0.61	0.039	2.34±0.34	2.63±0.48	0.791

Comparison between groups with independent sample t-test. Values presented as mean±SD. ESM, electronic supplementary material; CWD, children with type 1 diabetes; HC, healthy controls; SBP, systolic BP; DBP, diastolic BP; eGFR, estimated glomerular filtration rate; HDL-high density lipoprotein cholesterol; LDL, low density lipoprotein cholesterol

ESM table 2. Cardiac autonomic regulation at baseline with groups stratified by sex, children with type 1 diabetes compared to healthy controls.

ESM table 2. Baroreceptor sensitivity, baseline data with groups stratified by sex						
	**Girls CWD n=21**	**Girls HC n=16**	**p-value**	**Boys CWD n=19**	**Boys HC n=17**	**p-value**
**BRS-slope (ms/mmHg)**	20.1±7.2	18.5±7.1	0.538	18.2±8.4	20.4±7.2	0.408
**BRS z-score (SD)**	0.01±0.72	-0.15±0.71	0.538	-0.18±0.84	0.03±0.72	0.408
**QTVI**	−1.47±0.26	−1.55±0.20	0.361	−1.45±0.22	−1.42±0.29	0.728
**QTVI z-score (SD)**	0.08±0.79	-0.15±0.62	0.361	0.16±0.67	0.25±0.88	0.728
**HRV SDNN (ms)**	79.8±30.5	81.7±32.5	0.855	82.6±35.9	100.7±37.1	0.131

Comparison between groups with independent sample t-test. Values presented as mean±SD. ESM, electronic supplementary material; CWD, children with type 1 diabetes; HC, healthy controls; BRS, baroreceptor sensitivity; QTVI, QT variability index; HRV, heart rate variability

ESM table 3. Backwards multivariable regression on measures of cardiac autonomic regulation, significant models, *CWD and total study population*, respectively.

**ESM table 3. Backwards multivariable regression *on measures of cardiac autonomic regulation***				
**Children with type 1 diabetes**				
**QTVI**				
**Variables**	**Model 1**	**p-value**	**Model 2**	**p-value**
Age	0.049 (0.017)	0.0081	0.042 (0.015)	0.0100
Sex	−0.028 (0.064)	0.6702		
BMI z-score	0.004 (0.051)	0.9426		
Type 1 diabetes duration	−0.077 (0.025)	0.0048	−0.075 (0.021)	0.0013
HbA1c	0.011 (0.006)	0.0747	0.012 (0.005)	0.0326
SBP z-score	−0.076 (0.178)	0.6740		
DBP z-score	0.332 (0.213)	0.1294		
Cystatin C	−0.625 (0.246)	0.0168	−0.624 (0.236)	0.0125
**Intercept**	−1.548 (0.339)		−1.425 (0.285)	
**R-value**	0.726		0.693	
**R^2-value**	0.527		0.481	
**Model p-value**	0.0025		0.0002	
**HRV SDNN (ms)**				
**Variables**	**Model 1**	**p-value**	**Model 2**	**p-value**
Age				
Sex				
BMI z-score				
Type 1 diabetes duration				
HbA1c	−1.937 (0.848)	0.0283		
SBP z-score				
DBP z-score				
Cystatin C	64.869 (37.304)	0.0904		
**Intercept**	117.281 (45.766)			
**R-value**	0.390			
**R^2-value**	0.152			
**Model p-value**	0.0470			
**Total study population**				
**QTVI**				
**Variables**	**Model 1**	**p-value**	**Model 2**	**p-value**
**Age**	0.026 (0.013)	0.0576	0.030 (0.013)	0.0234
**Type 1 diabetes**	0.198 (0.120)	0.1055		
**Sex**	−0.063 (0.060)	0.3024		
**BMI z-score**	0.009 (0.040)	0.8186		
**HbA1c**	0.010 (0.007)	0.1478		
**SBP z-score**	0.197 (0.161)	0.2269	0.343 (0.138)	0.0161
**DBP z-score**	0.260 (0.204)	0.2078		
**Cystatin c**	−0.539 (0.242)	0.0302	−0.477 (0.240)	0.0512
**Intercept**	−2.130 (0.456)		−1.570 (0.232)	
**R-value**	0.477		0.391	
**R^2-value**	0.228		0.153	
**Model p-value**	0.0504		0.0158	
**HRV SDNN (ms)**				

Variables	Model 1	p-value	Model 2	p-value
**Age**	−2.081 (1.880)	0.2727		
**Type 1 diabetes**				
**Sex**	−7.059 (7.998)	0.3808		
**BMI z-score**				
**HbA1c**	−0.419 (0.451)	0.3559		
**SBP z-score**	−33.628 (21.567)	0.1239		
**DBP z-score**	−37.026 (27.260)	0.1792	−64.905 (22.205)	0.0047
**Cystatin c**	35.738 (33.866)	0.2953		
**Intercept**	142.635 (36.285)		120.836 (12.633)	
**R-value**	0.432		0.334	
**R^2-value**	0.187		0.112	
**Model p-value**	0.0369		0.0047	

First and simplest significant model from backwards multivariable regression, children with type 1 diabetes and total study population separately. Independent variables are listed below the dependent variable of each model. Values presented are beta coefficient. (standard error (SE)), R, R^2, Intercept

ESM, electronic supplementary material; HRV, heart rate variability; CV, coefficient of variation; SBP, systolic BP; DBP, diastolic BP
